# 

**Published:** 1906-07-07

**Authors:** 


CONTENTS.

**St. George's Hospital** - 241
**Treatment at Health Resorts** - 242
**Annotations** - 243
**Medical Opinion and Movement** - 244
**Hospital Clinics—**
Physical Culture and Spinal Curvature - 245
On the Practical Value of the Corneal Reflex in Examining the Eyes of Children - 247
**Practical Notes on Diagnosis and Treatment** - 248
**Remedies and their Uses** - 249
**Special Analytical and Health Commission on Tinned and Preserved Food** - 250
**The King Edward VII. Sanatorium** - 251
**New Appliances and Things Medical** - 251
**Medical Appointments and Vacancies** - 251
**Hospital Administration—**
St. Thomas's Hospital and Medical School - 252, 254
Current Hospital Topics - 253
Metropolitan Hospital - 254
Structural and Practical Departments - 255
Teaching Men How to Live - 255
**Editor's Letter-Box** - 255

*NURSING SECTION.*

**Notes on News from the Nursing World—**
The King and the Aged Nurse—The Secretary for War and the Army Nursing Service—Nursing the Injured at Salisbury—St. George's Hospital and Private Nursing—The New Matron of St. Mary's Hospital, Paddington—The New Maternity Department at the London Hospital—Night Nurses without Food—Three Years' Training at the Miller Hospital—Guardians and Discipline—A Reflection on the Court Suburb—The Pension Fund and Coventry Nurses—The Canon's Fear of a Nurse—Concert at St. Bartholomew's Hospital—The Examination of the Central Midwives Board—£10,000 for a Nursing Institution—St. Luke's Home for the Dying Poor—Short Items - 197-200
**The Nursing Outlook** - 201
**The Care and Nursing of the Insane** - 202
**The Nurse's Clinic** - 203
**Incidents in a Nurse's Life** - 204
**Central Midwives Board** - 205
**The Tuberculous of Paris** - 205
**North-Eastern Hospital for Children** - 206
**Everybody's Opinion** - 206
**Appointments** - 207
**Presentations** - 208
**The Nurses' Bookshelf** - 208
**A Book and Its Story** - 209
**Notes and Queries** - 210
**For Reading to the Sick** - 210

